# Respiratory safety and resource utilization in ERCP: a comparative study of high-flow nasal oxygen, conventional mask, and general anesthesia in high-risk populations

**DOI:** 10.3389/fmed.2026.1859787

**Published:** 2026-06-18

**Authors:** Kamil Taşkapılı, Emre Ballı, Mehlika Bilgi Kırmacı

**Affiliations:** 1 Department of Anesthesiology, Faculty of Medicine, Afyonkarahisar Health Sciences University, Afyonkarahisar, Türkiye; 2 Department of General Surgery, Faculty of Medicine, Afyonkarahisar Health Sciences University, Afyonkarahisar, Türkiye

**Keywords:** endoscopic retrograde cholangiopancreatography, general anesthesia, high-flow nasal oxygen, hypoxemia, procedural sedation

## Abstract

**Background:**

Endoscopic retrograde cholangiopancreatography (ERCP) poses significant respiratory challenges due to shared airways, prone positioning and deep sedation requirements. High-flow nasal oxygen (HFNO) has emerged as a potential non-invasive oxygenation strategy. This study compared the clinical efficacy and volumetric oxygen consumption of HFNO with conventional simple mask (SM) oxygenation and general anesthesia (GA) in ERCP procedures.

**Methods:**

This retrospective cohort study included 194 adult patients (SM: *n* = 67, HFNO: *n* = 56, GA: *n* = 71) who underwent ERCP between May 2025 and January 2026. Primary outcomes were desaturation incidence (SpO₂ < 92%) and volumetric oxygen consumption. Secondary outcomes included minimum SpO₂, airway interventions, and normalized propofol dose requirements. Subgroup analyses were performed for elderly (≥65 years) and ASA III patients.

**Results:**

Desaturation occurred in 59.7% of SM patients versus 0% in both HFNO and GA groups (*p* < 0.001). In elderly patients (*n* = 119), desaturation reached 81% in SM versus 0% in HFNO and GA. Minimum SpO₂ was significantly lower in SM (90%) compared to HFNO (99%) and GA (99%) (*p* < 0.001). Airway interventions were required in 31.4% of SM patients, 5.4% of HFNO patients (*p* = 0.004). Oxygen consumption was highest in HFNO (500 L) compared to SM (175 L) and GA (34.5 L) (*p* < 0.001). To ensure an objective comparison, propofol requirements were statistically analyzed using weight-and-time-normalized values (mg/kg/h); the significantly lower demand in the GA group was attributed to the anesthetic-sparing effect of volatile maintenance, whereas no significant difference was found between the two sedation-based groups (*p* = 0.110).

**Conclusion:**

HFNO provided a high level of respiratory stability, matching the observed 0% desaturation rate of the GA group, even in high-risk populations. While the anesthetic paradigms differ fundamentally, our findings suggest that HFNO can achieve a safety profile that approximates the clinical outcomes of general anesthesia regarding oxygenation maintenance.

## 1 Introduction

Endoscopic retrograde cholangiopancreatography (ERCP) is a complex therapeutic procedure requiring deep sedation or general anesthesia (1). However, the procedure carries significant anesthetic challenges due to the prone or semi-prone positioning of patients, shared airway with the endoscopist, and the requirement for a deep level of sedation, which often leads to dose-dependent respiratory depression (2).

Traditional oxygen delivery methods, such as simple face masks (SM) or standard nasal cannula, often fail to maintain adequate oxygenation during periods of apnea or hypoventilation induced by sedation (3). Consequently, general anesthesia (GA) with endotracheal intubation is frequently utilized to secure the airway, especially in complex cases. Although GA provides a definitive airway, it necessitates neuromuscular blockade and invasive instrumentation, which may not be optimal for all patient cohorts, particularly those with marginal physiological reserves (4).

High-flow nasal oxygen (HFNO) has redefined non-invasive ventilation by offering ‘apneic oxygenation’ a physiological bridge that maintains gas exchange during periods of reduced respiratory drive (5). This technique not only provides a PEEP-like effect to prevent alveolar collapse but also ensures effective CO2 washout from the anatomical dead space (6). Given its ability to maintain stable oxygenation levels even during periods of hypoventilation, HFNO is increasingly recognized as a vital tool in preventing desaturation during upper gastrointestinal endoscopy (7). While HFNO offers a robust non-invasive bridge to superior oxygenation, GA with endotracheal intubation remains the gold standard for absolute airway safety, particularly in patients with complex procedural requirements (8).

The decision-making process between these modalities involves a critical trade-off: the invasive safety of GA versus the non-invasive efficiency of HFNO. While the benefits of HFNO in endoscopy are documented, the trade-off between its superior respiratory safety and its significantly higher volumetric oxygen consumption (VO_2_) remains poorly quantified, especially when compared directly to the efficiency of semi-closed circuit general anesthesia. This study aims to fill this gap by evaluating whether the clinical benefits of HFNO (specifically in high-risk populations) justify its resource utilization. We hypothesized that HFNO would provide a clinical respiratory stability profile that is comparable to the outcomes observed in the GA group, while its higher oxygen consumption would be clinically justified by its efficacy in preventing hypoxemic events among high-risk patients.

## 2 Materials and methods

### 2.1 Study design and setting

This retrospective, single-center cohort study was conducted at the Endoscopy Unit of Afyonkarahisar Health Sciences University Hospital, a tertiary-care academic medical center in Turkiye. We reviewed medical records of adult patients who underwent elective ERCP procedures between May 2025 and January 2026. This nine-month study duration was selected to obtain a representative sample of ERCP cases while maintaining consistency in procedural protocols and anesthesia team composition.

### 2.2 Ethical approval and patient consent

The study protocol was approved by the Afyonkarahisar Health Sciences University Clinical Research Ethics Committee (approval date and number: 06 February 2026, 2026/2). The ethics committee granted a waiver of informed consent based on the following justifications: (1) the study involved retrospective analysis of existing medical records with no patient contact or intervention; (2) all oxygenation strategies evaluated were standard-of-care techniques routinely used in clinical practice at our institution; (3) patient data were de-identified before analysis to protect confidentiality; and (4) the study posed no more than minimal risk to participants. The study was conducted in full accordance with the ethical principles of the Declaration of Helsinki (2013 revision) and complied with local data protection regulations.

### 2.3 Patient selection and study population

#### 2.3.1 Inclusion criteria

Adult patients (aged 18–80 years) with American Society of Anesthesiologists (ASA) physical status classification I, II, or III who underwent elective ERCP were eligible for inclusion. All patients had an appropriate fasting status (at least 6 h for solid foods and at least 2 h for clear liquids) and provided written informed consent for the ERCP procedure and anesthesia care as part of routine clinical practice.

#### 2.3.2 Exclusion criteria

Patients were excluded from participation if any of the following criteria were present: (1) emergency ERCP procedures; (2) hemodynamic instability at baseline, defined as systolic blood pressure <90 mmHg or >180 mmHg, or heart rate <50 or >120 beats per minute; (3) severe pulmonary disease, defined as Global Initiative for Chronic Obstructive Lung Disease (GOLD) stage III or IV chronic obstructive pulmonary disease, or baseline oxygen saturation <90% on room air; (4) inadequate fasting duration; (5) body mass index (BMI) > 40 kg/m^2^; (6) pregnancy; or (7) incomplete medical or anesthetic records precluding accurate data extraction.

#### 2.3.3 Group assignment methodology

The choice of oxygenation strategy was determined by the attending anesthesiologist based on patient-specific risk factors, procedural complexity and institutional clinical pathways. Although not randomized, the anesthetic approach was standardized according to the patient’s ASA physical status and predicted procedural duration to minimize selection bias.

### 2.4 Oxygen delivery protocols

All oxygenation strategies were implemented according to standardized institutional protocols developed by our anesthesiology department. Detailed specifications for each method are provided below.

#### 2.4.1 SM group

Patients in the SM group received supplemental oxygen via a standard disposable simple face mask. During the induction phase, the mask was positioned to cover both the nose and mouth with an adequate seal. Once adequate sedation depth was achieved and the bite block was placed for duodenoscope insertion, the face mask was repositioned to cover the nasal passages only (from the nasal bridge to the upper lip). This modification allowed unobstructed oral access for the endoscope while maintaining continuous nasal oxygen delivery throughout the procedure. Oxygen was delivered at a flow rate of 5–8 liters per minute (L/min) with a FiO_2_ of 1.0 (100% oxygen). The mask was secured by the attending anesthesiologist and was returned to the standard nose-and-mouth position only after procedure completion and endoscope removal. The mask was not removed at any point during the procedure; only its position was modified to accommodate oral instrumentation.

#### 2.4.2 HFNO group

Patients received heated and humidified oxygen via the Optiflow THRIVE™ (Fisher & Paykel Healthcare) system. The flow rate was initiated and titrated up to 70 L/min with a FiO2 of 1.0. The nasal cannula was positioned with prongs inserted approximately 1 cm into the nares, secured with an adjustable strap around the head to prevent displacement during patient positioning. The HFNO system was initiated at least 3 min prior to sedation induction to allow for pre-oxygenation and patient acclimatization. Flow rate and FiO₂ settings remained constant throughout the procedure and were not adjusted unless an equipment malfunction occurred. The heated humidification system maintained gas temperature at 37 °C, measured at the distal end of the breathing circuit via an integrated thermistor.

#### 2.4.3 GA group

Patients in this group underwent general anesthesia with a definitive airway secured via endotracheal intubation and received controlled mechanical ventilation. Mechanical ventilation settings: volume-controlled ventilation (VCV), 6–8 mL/kg per ideal body weight, 10–12 breaths per minute, end-tidal CO₂ (EtCO₂) 35–40 mmHg, positive end-expiratory pressure (PEEP): 5 cmH₂O, FiO_2_: 0.40 (40%), inspiratory-to-expiratory (I:E) ratio: set to 1:2. Cases managed with supraglottic airway devices (e.g., LMA) were excluded from the study to ensure a standardized comparison of invasive vs. non-invasive techniques.

### 2.5 Anesthesia and sedation protocols

#### 2.5.1 Sedation protocol (SM and HFNO groups)

Although European Society of Gastrointestinal Endoscopy (ESGE) guidelines often highlight the efficacy of propofol monosedation, our institutional protocol favors a balanced sedation strategy using a combination of fentanyl and propofol. This approach was chosen to provide preemptive analgesia for the visceral pain associated with duodenal maneuvers and biliary cannulation during ERCP, which propofol alone does not address. Furthermore, the addition of a low-dose opioid allows for a ‘propofol-sparing effect,’ potentially reducing the total dose of propofol required to achieve deep sedation.

Opioid administration was strictly standardized across all sedation-based groups to minimize confounding respiratory depression. Sedation was initiated with a weight-based bolus of intravenous fentanyl (0.5–1.0 μg/kg). To prevent cumulative respiratory depression, additional fentanyl boluses (25–50 μg) were restricted to cases of inadequate analgesia (e.g., patient movement) and were administered at least 10 min apart. The total cumulative dose of fentanyl was recorded for each patient to ensure statistical comparability between the SM and HFNO cohorts. Maintenance of deep sedation was achieved with intermittent intravenous propofol boluses administered to maintain a Ramsay Sedation Scale (RSS) score of 5–6. The propofol dose was titrated to achieve deep sedation while preserving spontaneous ventilation. The target sedation level was loss of verbal response with maintained spontaneous respiratory effort. Propofol boluses were administered at a minimum 2-min intervals to allow for peak effect assessment. The total propofol dose administered during the procedure was recorded. To ensure an objective comparison of anesthetic depth between groups and to account for variations in procedural duration and patient weight, these doses were subsequently normalized and expressed as mg/kg/h for statistical analysis.

#### 2.5.2 General anesthesia protocol (GA group)

Patients in the GA group underwent standardized intravenous induction using propofol (1.5–2.5 mg/kg) and fentanyl (1–2 μg/kg). Neuromuscular blockade was managed with rocuronium (0.6 mg/kg) to allow for endotracheal intubation with a cuffed tube. Anesthesia was maintained with volatile anesthetics (sevoflurane or desflurane) at a MAC of 0.8–1.0. At procedure completion, volatile anesthetics were discontinued, FiO_2_ was increased to 1.0, and neuromuscular blockade was reversed with sugammadex (2 mg/kg IV). Extubation was performed when the patient met standard extubation criteria (adequate spontaneous ventilation, protective airway reflexes, response to verbal commands). Similar to the sedation groups, the total propofol used for induction in the GA group was recorded and normalized for analysis. It should be noted that the maintenance of anesthesia in this group was achieved via volatile anesthetics, which exerted a significant propofol-sparing effect compared to the total intravenous requirements in the sedation groups.

### 2.6 Intraoperative monitoring

All patients received standardized monitoring in accordance with ASA Basic Anesthetic Monitoring Standards. Pulse oximetry (SpO₂), electrocardiography (ECG), and non-invasive blood pressure (NIBP) measurements were recorded. Intermittent respiratory rate (visually assessed and documented every 5 min in both SM and HFNO groups) and depth of sedation (assessed every 5 min using the Ramsay Sedation Scale in both SM and HFNO groups) were evaluated. To ensure data integrity and avoid false-positive recordings, SpO_2_ was monitored continuously using high-sensitivity pulse oximetry. Clinical desaturation was strictly defined as a sustained drop in oxygen saturation below 92% for more than 15 s. Transient fluctuations related to probe displacement or motion artifacts were strictly excluded from the analysis. To further validate the 0% desaturation rates observed in the HFNO and GA groups, all electronic monitor data were cross-referenced with intraoperative nursing documentation to ensure that no transient hypoxemic events were overlooked or omitted.

### 2.7 Airway intervention

All airway interventions were documented in real-time by the anesthesia provider, including the type of intervention, timing, and patient response. For the purposes of analysis, airway interventions were categorized as manual maneuvers (jaw thrust/chin lift), bag-mask ventilation, or unplanned endotracheal intubation. In the GA group, airway management was prophylactic via endotracheal intubation; therefore, these patients were excluded from the analysis of ‘rescue manual airway interventions’ to ensure a valid comparison between non-invasive strategies (SM and HFNO).

### 2.8 Volumetric oxygen analysis

The primary economic parameter was the estimated gross oxygen delivery per patient, calculated using the following model:


$$\begin{array}{c} {\mathrm{VO}}_{2}\;(\text{liters})=\text{Flow rate}\;(L/\min)\times {\mathrm{FiO}}_{2}\\ \times \text{Total anesthesia duration}\;(\text{minutes}) \end{array}$$

This mathematical model was utilized as a standardized clinical tool to estimate gross oxygen delivery rather than precise metabolic consumption. While semi-closed circuits in the GA group allow for gas rebreathing, the model provides a consistent metric for comparing total resource allocation across different delivery systems.

### 2.9 Outcome definitions

The primary clinical outcome of this study was the incidence of respiratory compromise, specifically focused on hypoxemic events. The threshold for desaturation was defined as SpO_2_ < 92%, in accordance with the clinical criteria established by Khanna et al., identifying this level as a clinically significant indicator of respiratory distress during procedural sedation (7).

The secondary outcomes of this study were minimum SpO_2_ (the lowest SpO_2_ value recorded during the procedure, expressed as a percentage), airway intervention requirement (Categorized as none, jaw thrust/chin lift, bag-mask ventilation, or unplanned endotracheal intubation), total propofol requirements (the cumulative dose of propofol administered during the procedure), initially recorded in milligrams and subsequently normalized to patient weight and procedure duration (mg/kg/h) for statistical comparison, volumetric oxygen consumption (total volume of oxygen consumed during the procedure), the Charlson Comorbidity Index (CCI) along with the presence of chronic pulmonary comorbidities, 10-day all-cause mortality (death from any cause occurring within 10 days following the ERCP procedure, ascertained through review of hospital records and national death registry data). This outcome was included as an exploratory safety endpoint given the high-risk nature of the patient population undergoing ERCP.

### 2.10 Sample size and statistical power

Due to the retrospective cohort design of the study, no *a priori* sample size calculation was performed, and all eligible patients who underwent ERCP during the study period (May 2025 to January 2026) were included in the study. However, we conducted a *post-hoc* power analysis to assess the adequacy of our sample size.

The sample size calculation was guided by the prospective trial protocol described by Thiruvenkatarajan et al., in which a target enrollment of 132 patients (66 per treatment arm) was determined to yield 80% power for detecting a 16% reduction in hypoxic events during endoscopic retrograde cholangiopancreatography (ERCP) with HFNO compared to standard oxygen therapy (9). The present study, with 194 patients across three groups (SM: 67, HFNO: 56, GA: 71), exceeds this established benchmark.

To confirm adequate power for our specific design, we performed a *post-hoc* power analysis using G*Power 3.1. For the primary outcome (desaturation incidence), we calculated an effect size (w) of 0.70 based on the observed chi-square statistic (*χ*^2^ = 95.5, df = 2, *p* < 0.001). With *α* = 0.05 and *n* = 194, the achieved power was >99%, indicating that our sample size was more than adequate to detect the large effect observed in desaturation rates across the three groups.

### 2.11 Statistical analysis

Statistical analysis was performed using IBM SPSS Statistics for Windows, Version 25.0 (Armonk, NY: IBM Corp.). The normality of the data distribution was assessed using the Kolmogorov–Smirnov test and the Shapiro–Wilk test. Continuous variables are presented as median (interquartile range [IQR]) due to non-normal distribution, while categorical variables are expressed as frequencies and percentages (%). For continuous variables, the Kruskal–Wallis test was used to compare the three study groups. Categorical variables were analyzed using the Pearson Chi-square test. When a significant difference was detected among the three groups, pairwise *post-hoc* comparisons were performed using the Bonferroni test. A *p*-value of <0.05 was considered statistically significant for all analyses. For pairwise *post-hoc* Mann–Whitney U comparisons, rank-biserial correlation (*r*) was reported as the effect size measure. For pairwise categorical comparisons, odds ratios (OR) with 95% confidence intervals were calculated via Fisher’s exact test, and proportions were reported with 95% confidence intervals using the Wilson score interval method.

A multivariable logistic regression was conducted to adjust for confounding variables (age, BMI, ASA status). However, the model encountered complete separation due to the 0% event rate in the HFNO and GA cohorts. Consequently, while the model confirmed the groups as the primary predictor of SpO_2_ stability (Nagelkerke *R*^2^ = 1.0), stable adjusted odds ratios could not be calculated.

## 3 Results

**Figure 1:**
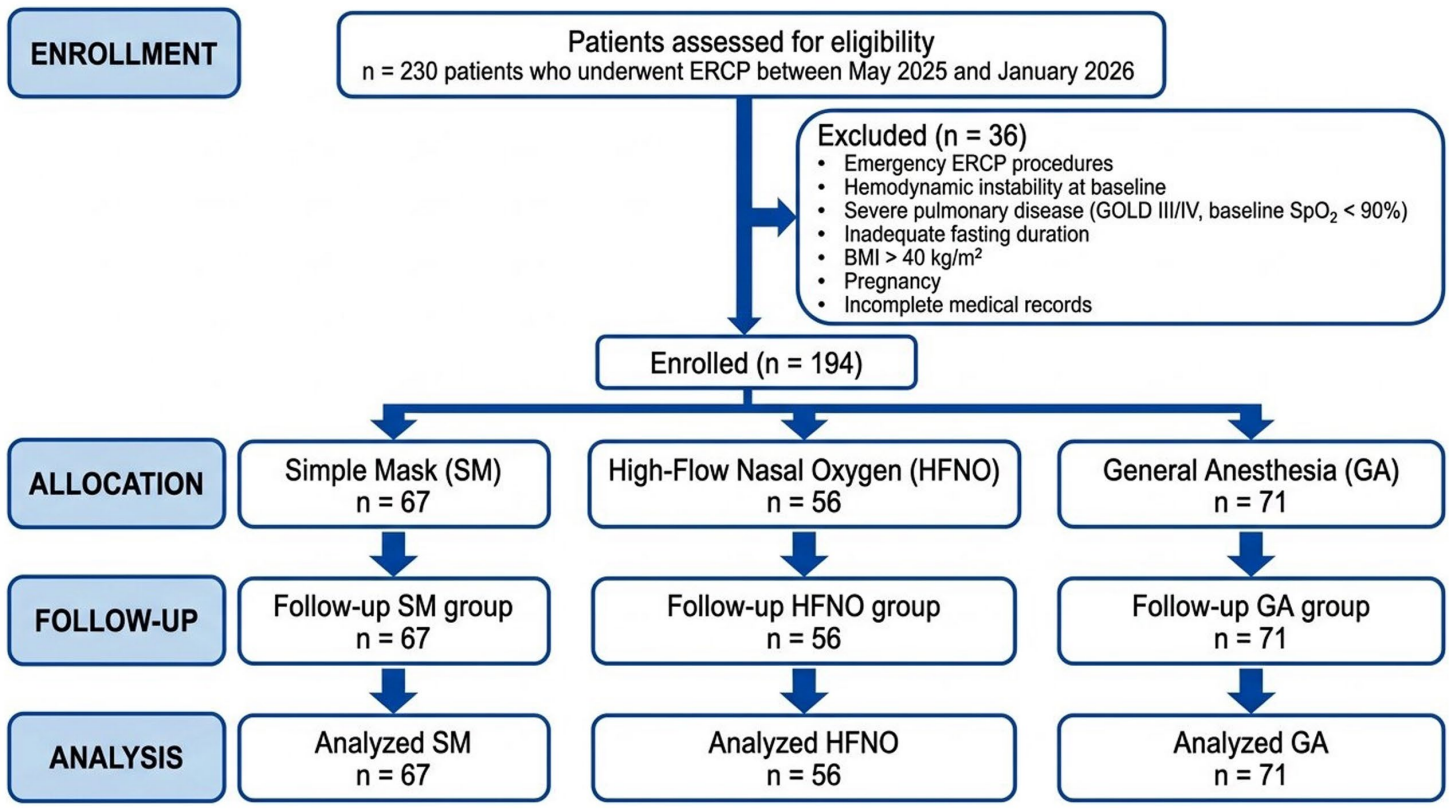
CONSORT flow chart describing participant progression through the study.

A total of 230 patients were screened for eligibility. After applying inclusion and exclusion criteria, 194 patients were included in the final analysis (Figure 1). The cohort was distributed into three groups based on the oxygenation strategy: SM (*n* = 67), HFNO (*n* = 56), and GA (*n* = 71). The demographic data, including age, sex, and BMI, were similar across the three groups (*p* = 0.941, *p* = 0.499, *p* = 0.636, respectively) (Table 1). There were no significant differences regarding ASA physical status or baseline oxygen saturation (SpO_2_) levels (Table 1). To further assess baseline clinical complexity and chronic disease burden, the Charlson Comorbidity Index (CCI) and specific comorbidities were evaluated. The median CCI scores were well-balanced among the groups (*p* = 0.226) and no significant disparity was found regarding the prevalence of pulmonary comorbidities (*p* = 0.493). Furthermore, the distribution of primary ERCP indications (including choledocholithiasis, malignant biliary obstruction, benign biliary strictures, and other benign causes) demonstrated strict homogeneity across the cohorts (*p* = 0.980) (Table 1). The median procedure duration was comparable between the groups, ranging from 22 to 24.5 min (*p* = 0.072), ensuring a standardized basis for normalized anesthetic and volumetric gas analysis (Table 1).

**Table 1:** Demographic and baseline clinical characteristics of the study population.

Parameter	SM (*n* = 67)	HFNO (*n* = 56)	GA (*n* = 71)	*p*-value
Age (years), median (IQR)	70 (22)	68.5 (25)	69 (22)	0.941^*^
Sex, *n* (%)				
Male	28 (38.4)	22 (30.1)	23 (31.5)	0.499^#^
Female	39 (32.2)	34 (28.1)	48 (39.7)	
BMI (kg/m^2^), median (IQR)	24.2 (3.8)	25.05 (3.3)	24.6 (4.5)	0.636^*^
Pulmonary comorbidity, *n* (%)	17 (34.7)	17 (34.7)	15 (30.6)	0.493^**#**^
ASA status, *n* (%)				
I	10 (26.3)	16 (42.1)	12 (31.6)	0.166^#^
II	27 (38)	14 (19.7)	30 (42.3)	
III	30 (35.3)	26 (30.6)	29 (34.1)	
Charlson Comorbidity Index	4 (3)	3.5 (4)	4 (4)	0.226^*^
ERCP indication, *n* (%)				
Choledocholithiasis	47 (34.1)	41 (29.7)	50 (36.2)	0.980^**#**^
Malignant biliary obstruction	5 (35.7)	4 (28.6)	5 (35.7)	
Benign biliary stricture	8 (32)	6 (24)	11 (44)	
Other benign causes	7 (41.2)	5 (29.4)	5 (29.4)	
Procedure duration (min), median (IQR)	22 (5)	24.5 (8)	23 (4)	0.072^*^
Baseline SpO2 (%), median (IQR)	95 (2)	94.5 (3)	94 (3)	0.106^*^

Data are expressed as number of patients (%), median (interquartile range). ^*^Kruskal–Wallis, ^#^Chi-square. BMI, body mass index; ASA, American Society of Anesthesiologists; ERCP, Endoscopic Retrograde Cholangiopancreatography; SpO_2_, oxygen saturation; SM, simple mask; HFNO, high-flow nasal oxygen; GA, general anesthesia.

The minimum SpO_2_ recorded during the procedure was significantly higher in the HFNO (99%) and GA (99%) groups compared to the SM group (90%) (*p* < 0.001) (Table 2). *Post-hoc* analysis confirmed that HFNO provided superior oxygenation over SM (*p* < 0.001), while showing no significant difference when compared to GA (*p* = 0.872) (Table 3). The overall effect size for this difference was large (ε^2^ = 0.684). Pairwise *post-hoc* analysis demonstrated a near-complete separation between SM and HFNO (*r* = 0.987, *p* < 0.001) and SM and GA (*r* = 0.997, *p* < 0.001), while no clinically meaningful difference was observed between HFNO and GA (*r* = 0.160, *p* = 0.872 after Bonferroni correction), consistent with the identical 99% median SpO₂ recorded in both groups.

**Table 2:** Comparison of clinical outcomes and volumetric oxygen consumption among the study groups.

Outcome parameter	SM (*n* = 67)	HFNO (*n* = 56)	GA (*n* = 71)	P-value
Minimum SpO2 (%), median (IQR)	90 (8)	99 (1)	99 (2)	<0.001^*^
Desaturation (SpO2 < 92%), *n* (%)	40 (59.7) [95% CI: 47.7–70.6]	0 (0) [95% CI: 0–6.4]	0 (0) [95% CI: 0–5.1]	<0.001^#^
Airway intervention, *n* (%)				
None	46 (68.6)	53 (94.6)	N/A (Endotracheal Intubation)	0.004^#^
Jaw thrust, chin lift	18 (26.9)	3 (5.4)		
Mask-balloon	1 (1.5)	0 (0)		
Intubation	2 (3)	0 (0)		
Oxygen consumption (L), median (IQR)	175 (44)	500 (257.5)	34.5 (6)	<0.001^*^
Propofol dose (mg), median (IQR)	170 (50)	180 (40)	120 (50)	<0.001^*^
Mortality, *n* (%)	8 (11.9) [95% CI: 6.2–21.8]	1 (1.8) [95% CI: 0.3–9.4]	1 (1.4) [95% CI: 0.2–7.6]	0.008^#^

Data are expressed as number of patients (%), median (interquartile range). ^*^Kruskal–Wallis, ^#^Chi-square. SpO_2_, oxygen saturation; SM, simple mask; HFNO, high-flow nasal oxygen; GA, general anesthesia.

**Table 3:** *Post-hoc* comparison results.

Outcome parameter	SM-HFNO	HFNO-GA	SM-GA
Minimum SpO2 (%), *p*-value*	<0.001	0.872	<0.001
Oxygen consumption (L), *p*-value*	<0.001	<0.001	<0.001
Propofol dose (mg), *p*-value*	0.110	<0.001	<0.001

^*^Bonferroni adjusted *p*-values. SM, simple mask; HFNO, high-flow nasal oxygen; GA, general anesthesia.

The incidence of hypoxemic events (SpO_2_ < 92%) was 59.7% (*n* = 40) in the SM group, whereas no desaturation events were observed in the HFNO and GA groups (*p* < 0.001) (Table 2). Consequently, airway interventions were significantly more frequent in the SM group (*n* = 21) compared to the HFNO group (*n* = 3) (*p* = 0.004). Patients in the GA group were excluded from this specific comparison as they were managed with prophylactic endotracheal intubation (Table 2). As no desaturation events were observed in either the HFNO or GA groups, a conventional odds ratio could not be estimated due to complete separation. The absolute risk difference between SM and HFNO was 59.7 percentage points (95% CI for SM: 47.7–70.6%; 95% CI for HFNO: 0–6.4%), and between SM and GA was 59.7 percentage points (95% CI for GA: 0–5.1%), each *p* < 0.001 by Fisher’s exact test. Patients in the SM group were significantly more likely to require any form of airway intervention compared to the HFNO group (OR = 8.07, 95% CI: 2.26–28.79, *p* = 0.0002 by Fisher’s exact test).

VO_2_ showed significant variability between the modalities (*p* < 0.001). The median was highest in the HFNO group (500 L), followed by the SM group (175 L), and lowest in the GA group (34.5 L) (Table 2). Pairwise comparisons indicated that all groups differed significantly from one another in terms of oxygen consumption (*p* < 0.001) (Table 3). The overall effect size was very large (ε^2^ = 0.850), indicating that the oxygenation modality was the dominant determinant of volumetric oxygen delivery. All pairwise comparisons demonstrated large effect sizes (rank-biserial *r*: SM vs. HFNO = 1.000; SM vs. GA = −0.972; HFNO vs. GA = −0.976; all *p* < 0.001 after Bonferroni correction).

To ensure an objective comparison of anesthetic depth, total propofol requirements were normalized to body weight and procedure duration. The median weight-and-time-adjusted propofol dose significantly differed across the groups (*p* < 0.001) (Table 2). Pairwise comparisons revealed that propofol consumption was significantly lower in the GA group (median: 120 mg) compared to both the HFNO (median: 180 mg) and SM (median: 170 mg) groups (*p* < 0.001 for both) (Table 3). This difference is attributed to the use of volatile anesthetics for maintenance in the GA group, which exerted a propofol-sparing effect. The overall effect size for propofol dose was large (ε^2^ = 0.405). Pairwise comparisons confirmed large effects for GA versus HFNO (*r* = −0.830, *p* < 0.001) and GA versus SM (*r* = −0.672, *p* < 0.001), while the difference between the two sedation-based groups was small and non-significant (SM vs. HFNO: *r* = 0.253, *p* = 0.110 after Bonferroni correction).

In contrast, no significant difference was observed in propofol requirements between the two sedation-based groups (HFNO vs. SM, *p* = 0.110) (Table 3). Furthermore, no significant difference was found between groups in fentanyl doses (*p* > 0.05), indicating a standardized baseline for opioid and benzodiazepine administration. This uniformity confirms that the observed differences in desaturation rates were not driven by disparities in opioid-induced respiratory depression but were primarily related to the oxygenation strategy employed.

The overall 10-day postoperative mortality rate showed a statistically significant disparity between the groups (*p* = 0.008); however, given the limited number of events, these results should be interpreted as exploratory findings. Pairwise comparisons revealed that the SM group had significantly higher odds of 10-day mortality compared to GA (OR = 9.49, 95% CI: 1.15–78.10, *p* = 0.015) and a trend toward higher mortality compared to HFNO (OR = 7.46, 95% CI: 0.90–61.58, *p* = 0.039), with the latter not reaching conventional significance thresholds given the small number of events. These findings are presented strictly as exploratory. In the elderly subgroup (Table 4), this gap was even more pronounced; 16.7% (*n* = 7) of the patients in the SM group died within 10 days, compared to 2.9% (*n* = 1) in the HFNO group and 2.3% (*n* = 1) in the GA group (*p* = 0.021). While the primary causes of death were associated with advanced malignancies and sepsis, the clustering of mortality in the SM group is an exploratory finding that warrants cautious interpretation, as no causal relationship can be established due to the study design.

**Table 4:** Subgroup analysis of patients over 65 years of age.

Outcome parameter	SM (*n* = 42)	HFNO (*n* = 34)	GA (*n* = 43)	*p*-value
Desaturation (SpO2 < 92%), *n* (%)	34 (81) [95% CI: 66.7–90.0]	0 (0) [95% CI: 0–10.2]	0 (0) [95% CI: 0–8.2]	<0.001^#^
Airway intervention, *n* (%)				
None	26 (61.9)	31 (91.2)	N/A (Endotracheal intubation)	<0.001^#^
Jaw thrust, chin lift	14 (33.3)	3 (8.8)		
Mask-balloon	1 (2.4)	0 (0)		
Intubation	1 (2.4)	0 (0)		
Oxygen consumption (L), median (IQR)	192 (36)	600 (250)	36 (7.5)	<0.001^*^
Propofol dose (mg), median (IQR)	150 (40)	165 (30)	110 (20)	<0.001^*^
Mortality, *n* (%)	7 (16.7) [95% CI: 8.3–30.6]	1 (2.9) [95% CI: 0.5–14.9]	1 (2.3) [95% CI: 0.4–12.1]	0.021^#^

Data are expressed as the number of patients (%), median (interquartile range). ^*^Kruskal–Wallis, ^#^Chi-square. SpO_2_, oxygen saturation; SM, simple mask; HFNO, high-flow nasal oxygen; GA, general anesthesia.

In the elderly subgroup (*n* = 119), the clinical advantage of HFNO became more pronounced. Desaturation (SpO_2_ < 92%) occurred in 81% of the SM group, while no desaturation events were observed in the HFNO or GA groups (*p* < 0.001) (Table 4). Airway interventions were required in 38.1% of the SM group (including manual maneuvers and unplanned intubations), compared to only 8.8% in the HFNO group. In this high-risk age group, volumetric oxygen consumption for HFNO reached a median of 600 L (Table 4).

For patients with significant systemic disease (ASA III, *n* = 85), HFNO and GA again demonstrated 0% desaturation rates, whereas the SM group continued to show high respiratory instability (*p* < 0.001) (Table 5). The demand for manual airway support in ASA III patients was significantly reduced in the HFNO group compared to the SM group (Table 5). The propofol requirements remained consistent with the general population, where significantly less propofol was used in the GA group due to the anesthetic-sparing effect of volatile gasses (*p* < 0.001).

**Table 5:** ASA 3 patient subgroup analysis.

Outcome parameter	SM (*n* = 30)	HFNO (*n* = 26)	GA (*n* = 29)	*p*-value
Desaturation (SpO2 < 92%), *n* (%)	29 (96.7) [95% CI: 83.3–99.4]	0 (0) [95% CI: 0–12.9]	0 (0) [95% CI: 0–11.7]	<0.001^#^
Airway intervention, *n* (%)				
None	12 (40)	23 (88.5)	N/A (Endotracheal intubation)	<0.001^#^
Jaw thrust, chin lift	15 (50)	3 (11.5)		
Mask-balloon	1 (3.3)	0 (0)		
Intubation	2 (6.7)	0 (0)		
Oxygen consumption (L), median (IQR)	192 (40)	695 (285)	36 (6.8)	<0.001^*^
Propofol dose (mg), median (IQR)	140 (33)	170 (35)	110 (20)	<0.001^*^
Mortality, *n* (%)	8 (26.7) [95% CI: 14.2–44.4]	1 (3.8) [95% CI: 0.7–18.9]	1 (3.4) [95% CI: 0.6–17.2]	0.007^#^

Data are expressed as the number of patients (%), median (interquartile range). ^*^Kruskal–Wallis, ^#^Chi-square. SpO_2_, oxygen saturation; SM, simple mask; HFNO, high-flow nasal oxygen; GA, general anesthesia.

## 4 Discussion

The management of the shared airway in ERCP remains a significant challenge for anesthesiologists. Our study highlights that HFNO serves as a potent non-invasive alternative that bridges the gap between conventional mask oxygenation and invasive general anesthesia.

Hypoxemia is the most common adverse event during procedural sedation for ERCP (10). In our study, the SM group had a 59.7% desaturation rate (SpO_2_ < 92%), necessitating frequent airway maneuvers. This is consistent with recent meta-analytic evidence demonstrating that HFNO significantly reduces the risk of hypoxia compared to conventional oxygen therapy across various endoscopic procedures (7). However, our study provides a unique perspective by demonstrating that HFNO achieved a 0% desaturation rate even under a stringent SPO_2_ < 92% threshold; a level of precision clinically comparable to GA. Furthermore, Liu et al. reported that HFNO reduced the incidence of hypoxia from 5.66 to 0% during gastrointestinal endoscopy (11). Although our desaturation rate in the SM group was higher due to the deeper sedation levels and prone positioning required for ERCP, the absolute success of HFNO in our cohort reinforces the findings of Hung et al., who identified HFNO as a superior rescue and maintenance strategy in high-risk gastrointestinal interventions (12). In our study, we adopted a SpO_2_ < 92% threshold for desaturation, a decision supported by the clinical sensitivity criteria established by Khanna et al. (7). While some studies in the literature use a more liberal cut-off of 90%, we selected 92% to provide a safer detection margin for early respiratory compromise, particularly crucial for our high-risk elderly and ASA III cohorts (9). This proactive threshold likely contributes to the higher incidence of desaturation recorded in the SM group but highlights the precision of HFNO in maintaining oxygenation even under more stringent safety criteria.

The desaturation (59.7% in SM) and exploratory 10-day mortality (11.9% in SM) rates reported in our study appear higher than those described in some contemporary ERCP literature, such as the recent study by Prosenz et al. (13). This discrepancy can be primarily attributed to our institutional profile and methodological rigor. As a specialized tertiary-care academic center, our cohort represents a significantly more complex and fragile patient population, evidenced by the high prevalence of elderly (61.3%) and ASA III (43.8%) individuals who often present with multiple co-existing pathologies. Furthermore, our stringent and proactive definition of desaturation (SpO_2_ < 92%) set a higher safety threshold compared to the more liberal limits (e.g., 90% or 85%) frequently used in other studies. This conservative approach allowed for the early detection of even subtle respiratory compromise, which naturally resulted in a higher recorded incidence of events in the SM group but also highlights the superior protective capacity of HFNO in such high-risk settings.

The physiological mechanisms underlying HFNO’s superior performance include the generation of positive end-expiratory pressure (PEEP) of 2–7 cmH₂O, which prevents alveolar collapse and maintains functional residual capacity (FRC) during sedation-induced hypoventilation (7, 14). This PEEP effect is particularly critical in the prone position required for ERCP, where gravitational forces and abdominal compression further reduce FRC and promote atelectasis (15). Our results suggest that this PEEP effect provides enough of a “safety bridge” to maintain FRC even during the intermittent periods of hypoventilation induced by propofol boluses. Additionally, HFNO provides continuous washout of the nasopharyngeal dead space, reducing CO_2_ rebreathing and improving alveolar ventilation efficiency (16). We acknowledge that the absence of capnography in the sedation groups limits our ability to quantify CO_2_ retention and subclinical hypoventilation. However, previous physiological studies have demonstrated that the high flow rates used in HFNO effectively promote nasopharyngeal dead space washout, which facilitates CO_2_ clearance even during periods of reduced minute ventilation. Thus, while PaCO_2_ levels were not measured, the clinical stability observed in the HFNO group suggests effective alveolar ventilation.

This physiological buffer explains a key finding in our cohort: the 3 patients (5.4%) in the HFNO group who required manual airway maneuvers (jaw thrust) maintained a minimum SpO_2_ of 99% throughout the event. In these cases, the intervention was triggered by visual and clinical signs of upper airway obstruction (such as stridor or paradoxical respiratory effort) induced by deep sedation, rather than a response to falling oxygen levels. Due to the high-flow oxygen reservoir and apneic oxygenation capability inherent to the system, the anesthesiologist was able to correct the mechanical obstruction preemptively. This demonstrates that HFNO effectively decouples upper airway obstruction from immediate hypoxemic distress, providing a critical safety margin that allows clinicians to intervene before desaturation can occur.

Our subgroup analyses revealed that high-risk populations particularly benefit from HFNO. Elderly patients (≥65 years) in the SM group experienced 81% desaturation rates, while ASA III patients showed 96.7% desaturation rates. These findings are consistent with recent evidence demonstrating that elderly and high-risk patients undergoing ERCP have significantly reduced hypoxemia when managed with HFNO compared to conventional oxygen therapy (17). The age-related decline in functional residual capacity, respiratory muscle strength, and cardiovascular reserve makes these patients particularly vulnerable to desaturation during procedural sedation (18).

One of the most significant findings was that HFNO achieved an oxygenation profile that mirrored the results seen in the GA group. We acknowledge that HFNO (spontaneous ventilation) and GA (controlled mechanical ventilation) are fundamentally different anesthetic strategies and do not imply formal statistical equivalence or non-inferiority. However, from a practical clinical perspective, the prevention of desaturation by HFNO reached a level of precision typically associated with the secure airway of GA. While GA provides a definitive airway, it is inherently more invasive than HFNO and introduces potential risks associated with neuromuscular blockade and endotracheal intubation (19). The non-invasive nature of HFNO offers a comparable safety margin for many patients without the physiological stress of intubation (8).

Regarding our sedation regimen, we utilized a combination of fentanyl and propofol instead of propofol monosedation. While some guidelines, such as those from the ESGE, suggest propofol-based strategies, balanced sedation with opioids is widely utilized in clinical practice to manage procedural pain and improve patient tolerance during complex interventions like ERCP. By including fentanyl, we aimed to provide adequate analgesia and minimize the hemodynamic fluctuations sometimes associated with higher doses of propofol monosedation. The addition of fentanyl to propofol reflects a balanced sedation approach widely used in ERCP practice, supported by randomized controlled trials and a 2024 network meta-analysis demonstrating that propofol-opioid combinations improve periprocedural oxygenation and procedural conditions compared to propofol monosedation (20). This regimen was standardized across all three study groups, ensuring that pharmacological differences did not confound the primary comparison of oxygenation strategies.

We acknowledge that our oxygen utilization model is a simplified estimation of gross delivery and does not account for the metabolic efficiency of semi-closed GA circuits. In clinical practice, low-flow anesthesia significantly reduces actual oxygen consumption compared to the high-flow requirements of HFNO. Therefore, our health-economic inferences should be viewed as exploratory institutional resource estimates rather than definitive cost-effectiveness data. However, even with this simplified model, the disparity between the 500 L delivery in HFNO and the 34.5 L in GA highlights a significant trade-off between non-invasive stability and gas utilization efficiency.

Our exploratory analysis revealed a higher clustering of 10-day mortality in the SM group, particularly among high-risk elderly patients. While it is tempting to link these outcomes to repetitive desaturation events, we acknowledge that this finding is strictly hypothesis-generating. Importantly, the decision-making process for oxygenation strategy allocation was governed strictly by institutional logistics and sequential device availability, rather than patient prognosis or terminal status; no palliative or higher-risk patients were intentionally directed toward the SM cohort. This is methodologically validated by the strict homogeneity of baseline characteristics across all groups, including age, baseline SpO_2_, and ASA III distribution (*p* > 0.05), confirming a balanced baseline risk profile. These patients often suffered from advanced malignancies or sepsis, which are the primary drivers of mortality in this population. Due to the retrospective nature of our study and the lack of adjustment for frailty or malignancy burden, no causal link can be established between oxygenation strategy and survival. The higher physiological stress associated with frequent hypoxemia in the SM group might have contributed to a ‘second hit’ phenomenon, but this remains a subject for future well-powered prospective trials.

Our study has several limitations. First, the retrospective design introduces potential selection bias, as the choice of oxygenation strategy was not randomized and may have been influenced by patient characteristics or physician preference. Second, this is a single-center study, which may limit the generalizability of our findings to other institutions with different patient populations or practice patterns. Third, we lacked objective depth-of-anesthesia monitoring (e.g., BIS or Entropy), relying instead on validated clinical scales (RSS), which may not detect subtle variations in anesthetic depth. Fourth, oxygen delivery calculation is a simplified model for gross resource estimation and does not account for the efficiency of gas rebreathing or specific fresh gas flow settings in the GA group. Consequently, the economic implications are limited to institutional gas delivery logistics and do not constitute a full formal cost-effectiveness analysis. Fifth, we did not perform capnography monitoring, precluding detailed analysis of CO_2_ dynamics during HFNO use. Sixth, the SM group oxygenation technique involved mask repositioning from full-face to nasal-only coverage upon endoscope insertion. While this is a standard clinical practice at our institution, it introduces variability in the effective FiO₂ delivered during the intra-procedural phase. Seventh, we did not assess patient-reported outcomes such as comfort, satisfaction, or procedural recall, which are important considerations for patient-centered care. Eighth, the mortality data should be interpreted with caution, as deaths were likely related to underlying comorbidities rather than the oxygenation strategy itself. Finally, our economic analysis was simplified and did not include indirect costs such as productivity loss or long-term complications.

In conclusion, HFNO is a highly effective non-invasive strategy that provides a degree of respiratory safety that aligns with the results observed under general anesthesia, without the need for invasive airway management. While not a replacement for GA in cases requiring absolute airway protection, HFNO offers an alternative for maintaining oxygenation stability in high-risk patients undergoing deep sedation. Although HFNO requires higher volumetric oxygen consumption than conventional methods, its clinical benefits, specifically the complete prevention of desaturation and reduced requirement for manual airway interventions, justify this resource utilization, particularly in fragile populations such as elderly and ASA III patients.

While general anesthesia remains an efficient approach for gas conservation, HFNO offers a superior non-invasive alternative for high-risk patients, ensuring optimal oxygenation stability without the physiological stress of intubation. Our findings suggest that HFNO may serve as a viable non-invasive oxygenation strategy for elderly and high-risk patients undergoing ERCP under deep sedation. While it does not replace the definitive airway protection of GA, it offers an observed safety margin regarding oxygenation stability that merits consideration as an alternative to conventional mask oxygenation in complex procedural settings.

## Data Availability

The original contributions presented in the study are included in the article/supplementary material, further inquiries can be directed to the corresponding author.
